# Episodic Memory and Episodic Future Thinking Impairments in High-Functioning Autism Spectrum Disorder: An Underlying Difficulty With Scene Construction or Self-Projection?

**DOI:** 10.1037/neu0000005

**Published:** 2013-09-09

**Authors:** Sophie E. Lind, David M. Williams, Dermot M. Bowler, Anna Peel

**Affiliations:** 1 Department of Psychology, Durham University; 2 Department of Psychology, City University London

**Keywords:** autism spectrum disorder, episodic memory, episodic future thinking, scene construction, self-projection

## Abstract

***Objective:*** There appears to be a common network of brain regions that underlie the ability to recall past personal experiences (episodic memory) and the ability to imagine possible future personal experiences (episodic future thinking). At the cognitive level, these abilities are thought to rely on “scene construction” (the ability to bind together multimodal elements of a scene in mind—dependent on hippocampal functioning) and temporal “self-projection” (the ability to mentally project oneself through time—dependent on prefrontal cortex functioning). Although autism spectrum disorder (ASD) is characterized by diminished episodic memory, it is unclear whether episodic future thinking is correspondingly impaired. Moreover, the underlying basis of such impairments (difficulties with scene construction, self-projection, or both) is yet to be established. The current study therefore aimed to elucidate these issues. ***Method:*** Twenty-seven intellectually high-functioning adults with ASD and 29 age- and IQ-matched neurotypical comparison adults were asked to describe (a) imagined atemporal, non-self-relevant fictitious scenes (assessing scene construction), (b) imagined plausible self-relevant future episodes (assessing episodic future thinking), and (c) recalled personally experienced past episodes (assessing episodic memory). Tests of narrative ability and theory of mind were also completed. ***Results:*** Performances of participants with ASD were significantly and equally diminished in each condition and, crucially, this diminution was independent of general narrative ability. ***Conclusions:*** Given that participants with ASD were impaired in the fictitious scene condition, which does not involve self-projection, we suggest the underlying difficulty with episodic memory/future thinking is one of scene construction.

Recently, an important link has been made between the ability to mentally re-experience past episodes (episodic memory) and the ability to imagine episodes that one might plausibly experience in the future. This latter ability to mentally pre-experience possible future events has been termed “episodic future thinking” (Atance & O’Neill, 2001). Episodic memory and episodic future thinking emerge simultaneously in typical development (Suddendorf, 2010) and decline in parallel among older adults (Addis, Wong, & Schacter, 2008). Furthermore, individuals with acquired amnesia, who are unable to remember past personal experiences, show a corresponding deficit in imagining future personal experiences (Klein, Loftus, & Kihlstrom, 2002; Tulving, 1985). The same is true of individuals with psychiatric disorders, such as depression (Williams et al., 1996) or schizophrenia (D’Argembeau, Raffard, & Van der Linden, 2008), who show attenuated ability to generate both past personal experiences and possible future personal experiences. Together, these findings suggest that episodic memory and episodic future thinking may have a common underlying cognitive (and neurobiological) basis.

## Theories of the Underlying Link Between Episodic Memory and Episodic Future Thinking

According to one prominent theory, episodic memory and episodic future thinking are linked because both involve elements of self-awareness. In particular, Buckner and Carroll (2007) have argued that both require the capacity for “self-projection,” which they define as the ability to shift from one’s current perspective to alternative perspectives (temporal, spatial, or mental). This idea is similar to the idea originally proposed by Tulving (2005, for example), and developed by Suddendorf and Corballis (1997), that both episodic memory and episodic future thinking depend on “mental time travel” (the ability to mentally project oneself backward in time in order to reexperience past episodes or forward in time in order to preexperience future episodes) and involve autonoetic (self-knowing) consciousness. Buckner and Carroll have further suggested that self-projection also underpins the ability to attribute mental states to others (an aspect of theory of mind; Premack & Woodruff, 1978) and spatial navigation. They argue that episodic memory/episodic future thinking, theory of mind, and navigation are all forms of cognition that “rely on autobiographical information and are constructed as a perception of an alternative perspective or, in the case of theory of mind, a simulation that considers another individual’s perspective” (p. 49). Although cognitive-experimental evidence for this hypothesis is scarce, several studies have explored the relationship between episodic memory and theory of mind, and have observed a positive association between these abilities (e.g., Naito, 2003; Perner & Ruffman, 1995).

A second prominent theory, put forward by Hassabis and colleagues (e.g., Hassabis, Kumaran, & Maguire, 2007; Hassabis, Kumaran, Vann, & Maguire, 2007; Hassabis & Maguire, 2007; Hassabis & Maguire, 2009), suggests that episodic memory and episodic future thinking rely on a common underlying process of scene construction. Scene construction is the ability to mentally generate and maintain a coherent, *multimodal* spatial representation. Unlike simple visual imagery, which involves the mental generation and maintenance of a single element, scene construction involves *binding together* multiple elements of an imagined scene, including contextual details such as sounds, smells, feelings, thoughts, people, and objects. It is typically operationalized by asking participants to provide rich verbal descriptions of multimodal, *a*temporal and *im*personal fictitious scenes (e.g., a sandy beach in a tropical bay) generated in their mind’s eye.

**Figure 1 fig1:**
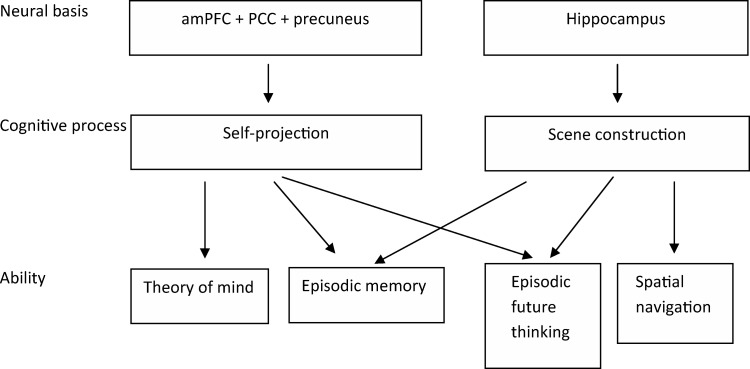
Schematic interpretation of the theory put forward by Hassabis, Kumaran, and Maguire (2007). amPFC = anterior medial prefrontal cortex; PCC = posterior cingulate cortex.

Hassabis et al. accept that temporal self-projection and self-related processing play a role in episodic memory and episodic future thinking. However, these processes are considered “add-ons” to the basic contribution to episodic memory and episodic future thinking of the ability to construct multimodal scenes in one’s mind. Specifically, Hassabis, Kumaran, and Maguire (2007, p. 14372) argue that episodic memory and episodic future thinking involve “at least two components with dissociable neural bases: a network centered on the hippocampus responsible for scene construction, with the amPFC [anterior medial prefrontal cortex], PCC [posterior cingulate cortex] and precuneus mediating self-projection in time, sense of familiarity, and self-schema” (see Figure 1 for a schematic depiction of this model).[Fig-anchor fig1]

Furthermore, Hassabis and Maguire (2007) argue that each of the multiple cognitive functions described by Buckner and Carroll (2007; episodic memory, episodic future thinking, theory of mind, and navigation) may rely to a greater or lesser extent on these subcomponents. For example, whereas navigation might rely exclusively upon the hippocampal (scene construction) system, theory of mind might rely *exclusively* on the frontal (self-related processing) system, and episodic memory and episodic future thinking might rely on both systems.

## Links Between Episodic Memory and Episodic Future Thinking: The Case of Autism Spectrum Disorder

Autism spectrum disorder (ASD) refers to a set of developmental disorders diagnosed on the basis of significant behavioral impairments in social interaction, communication, and behavioral flexibility (American Psychiatric Association, 2000; World Health Organization, 1993). At the cognitive level of description, ASD is characterized by a selective diminution of episodic memory, leaving semantic memory undiminished (e.g., Bowler, Gardiner, & Gaigg, 2007; Bowler, Gardiner, & Grice, 2000), as well as by impairments in theory of mind (e.g., Happé, 1995). If it is the case that episodic memory and episodic future thinking rely on the same underlying processes, as the theories outlined here suggest, then any disorder that involves deficits in one of the abilities should also involve deficits in the other ability. Given that ASD involves a well-established episodic memory deficit, episodic future thinking should also be impaired in this disorder, if the theories discussed are true. As such, the investigation of episodic future thinking ability in ASD provides a potential test of these theories.

To our knowledge, only two full studies have been published on episodic future thinking in ASD.^1^ Lind and Bowler (2010) adopted a method originally devised by D’Argembeau and Van der Linden (2004). Adults with ASD and closely age- and IQ-matched comparison adults were asked to recall seven specific events from particular time periods in the past (ranging from today to 10 years ago; episodic memory condition) and imagine seven events from corresponding time points in the future (ranging from today to in 10 years’ time; episodic future thinking condition), and give verbal descriptions of them. Descriptions were independently rated for quality and the specificity that would indicate true episodic memory/episodic future thinking. Lind and Bowler found that descriptions of *both* past and future events were significantly less specific and lower in quality among participants with ASD than among comparison participants, reflecting diminished episodic memory *and* episodic future thinking. This diminution was associated with a moderate to large effect size (*r* = .43).

Recently, Crane, Lind, and Bowler (2013) sought to assess episodic memory and episodic future thinking in ASD using a different method from that employed by Lind and Bowler (2010). This method involved a sentence completion task designed to elicit past and future event descriptions (cf. Raes, Hermans, Williams, & Eelens, 2007; see also Anderson & Dewhurst, 2009). Adults with and without ASD were presented with a series of stems such as “I still remember well how …” and “Next year I …” and were asked to complete the sentences. In contrast to Lind and Bowler’s findings (and the majority of research on episodic memory in ASD), Crane et al. did not find any group differences in either the past or future event conditions. Group differences in scores were all associated with effect sizes that were negligible or small (all Cohen’s *d*s < .32).

Although the results of Crane et al. (2013) are intriguing, it is distinctly possible that the failure to observe group differences in past or future thinking was an artifact of the particular measure used (as noted by the authors). Arguably, the sentence completion task was a somewhat insensitive measure of episodic memory and episodic future thinking. In particular, participants were not explicitly instructed to describe *specific* (i.e., episodic) events. Indeed, the sentence stems may not have directed participants from *either* group that they were supposed to produce specific event descriptions. Thus, any underlying group differences in episodic memory and episodic future thinking may have been masked by the fact that participants from both groups could rely purely on semantic knowledge to provide nonspecific (nonepisodic) descriptions. Nonetheless, these mixed findings suggest the need for further research. This need is further emphasized when considering the fact that no study of episodic future thinking in ASD has been directed at elucidating the underlying basis of any potential difficulty.

On the one hand, it is possible that both episodic memory and episodic future thinking deficits in ASD are explained by an underlying difficulty with basic scene construction (associated with hippocampal dysfunction). Certainly, this idea dovetails certain existing theories of the causes of episodic memory deficits in ASD. In particular, Bowler and colleagues (e.g., Bowler, Gaigg, & Lind, 2011) have suggested that a difficulty with “binding,” which involves encoding the relations between elements that comprise an episode into a single representation and which is associated with (anterior) the hippocampal functioning, plays a central role in producing the memory profile characteristic of ASD (Bowler, Gaigg, & Lind, 2011). Moreover, a problem with scene construction in ASD is also consistent with the suggestion that individuals with ASD have a perceptual/cognitive processing style that is characterized by “weak central coherence” (see Happé & Frith, 2006). According to this view, individuals with ASD tend not to process environmental stimuli as coherent wholes (global processing), but instead focus on each individual element (feature-based processing). As such, people with ASD tend not to “see the wood for the trees.” This tendency to focus on individual elements of environmental scenes may extend to, or even underlie, any potential difficulty with imagining coherent scenes *in mind*.

On the other hand, difficulties with episodic memory and episodic future thinking in ASD could be explained instead by a selective deficit in self-projection (associated with prefrontal cortex dysfunction). It may be that individuals with ASD are fully capable of forming coherent, multimodal representations of atemporal, non-self-related fictitious scenes (i.e., that people with ASD have intact scene construction ability), but have difficulty mentally projecting themselves through time to “identify with” a past state of self or an anticipated future state of self. In other words, the difficulty in ASD could be with self-projection/self-related processing, rather than with scene construction. This idea is consistent with the notion that ASD involves diminished awareness of aspects of self (e.g., Williams, 2010), as well as with theories that explicitly implicate diminished self-awareness as a contributory cause of the specific profile of memory that characterizes ASD (e.g., Lind, 2010).

To explore these issues, we employed a version of the experimental task developed by Hassabis, Kumaran, & Maguire (2007). To assess episodic future thinking ability, participants were asked to provide detailed descriptions of imagined specific events that they might plausibly experience in the future (possible future Christmas event, possible event over next weekend, possible future meeting with a friend/relative). To assess episodic memory ability, participants were also asked to describe memories of specific previously experienced past events (last birthday, last week, and last time they went shopping). Finally, to assess the ability to imagine atemporal, non-self-relevant fictitious scenes (i.e., scene construction ability), participants were asked to provide detailed descriptions of imagined commonplace settings (beach, market, ship, pub, forest, and museum).

Our rationale was that whereas all conditions of the task required basic scene construction ability, only the past and future events conditions of the task additionally required self-projection, because only these conditions involved imagining the self-relevant scenarios requiring mental time travel. As such, if episodic memory and episodic future thinking deficits in ASD are primarily due to a diminution of *self-projection*, then we should expect to see impaired performance among ASD participants in the past and future events conditions only. Alternatively, if episodic memory and episodic future thinking deficits in ASD are primarily the result of diminished *scene construction* ability, then the performance of participants with ASD should be equally impaired across all conditions. Of course, a final possibility is that difficulties with scene construction and difficulties with self-projection independently contribute to episodic future thinking and episodic memory deficits in ASD. In this case, participants with ASD should be impaired in all conditions, but relatively more so in the past and future events conditions.

We also included a narrative control task, which involved participants providing an ongoing narrative of a 24-page picture book, *Frog, Where Are You?* (Mayer, 1969). It is important to note that none of the studies of episodic future thinking cited above (in either ASD or other populations) included a measure of online narration ability (cf. Losh & Capps, 2003). As such, the group differences in experimental task performance reported in these studies could merely reflect general difficulties with narration, rather than specific difficulties with episodic future thinking. After all, performance on any experimental measure of episodic future thinking that involves giving complex verbal descriptions of imagined episodes could be diminished purely as a result of attenuated narrative ability (see Addis & Schacter, 2012; Gaesser, Sacchetti, Addis, & Schacter, 2011; Race, Keane, & Verfaellie, 2011).

Finally, we also employed the animations task (Abell, Happé, & Frith, 2000) as a measure of theory of mind ability. As discussed above, Buckner and Carroll (2007) argue that self-projection is critical for theory of mind in that (according to their view), comprehending another person’s mental state requires one to “shift from the present perspective to a simulated model of an alternative world” (Buckner & Carroll, 2007, p. 51). In contrast, Hassabis and Maguire (2007) argue that scene construction is not necessary for accurate theory of mind.^2^ On this basis, episodic future thinking ability and episodic memory ability might be associated significantly with theory of mind ability, whereas basic scene construction ability should not be. Notably, the majority of the evidence on which these theories are based is derived from neuroimaging studies. As far as we know, no study has explicitly investigated the association between episodic future thinking (or the processes of scene construction and self-projection that arguably underlie episodic future thinking) and theory of mind using cognitive-experimental methods. As such, this was an important aim of our study.

## Method

### Participants

Ethical approval for this study was obtained from the appropriate university ethics committee. Twenty-seven adults with high-functioning ASD (21 male; 6 female) and 29 neurotypical comparison adults (22 male; 7 female) took part in this experiment, after giving written, informed consent to take part. All participants were paid standard university fees for their participation. Participants with ASD were recruited (a) via an advertisement on the “Research Projects: Be a Participant” page of The National Autistic Society U.K. Web site (www.autism.org.uk), (b) through local ASD support groups, (c) through the Durham University Service for Students with Disabilities, and (d) through word of mouth. The majority of comparison participants were recruited through advertisements in local newspapers. However, a small number took part in order to receive course credits in partial fulfillment of their undergraduate psychology degrees. Inclusion criteria included having a full-scale IQ of at least 85, being aged 16 to 65 years, and having no neurological or psychiatric disorders other than ASD (no participants needed to be excluded for failing to meet these criteria). Participants in the ASD group had all received formal diagnoses of autistic disorder (*n* = 5) or Asperger’s disorder (*n* = 22), according to conventional criteria (American Psychiatric Association, 2000; World Health Organization, 1993). All documented diagnostic information was checked thoroughly and provided sufficient information to ensure diagnostic criteria were met in each case.

To assess severity of current ASD features among participants with ASD and the presence of ASD-like features among comparison participants, several measures were taken. First, participants themselves completed the Autism-spectrum Quotient (AQ; Baron-Cohen, Wheelwright, Skinner, Martin, & Clubley, 2001). The AQ is a 50-item questionnaire that is suitable for administration with adults whose intelligence is within the average or above-average range, and which provides a quantitative index of self-reported ASD traits. Only three participants with ASD missed the ASD cutoff on the AQ (26 points; Woodbury-Smith, Robinson, Wheelwright, and Baron-Cohen, 2005). All comparison participants scored below the ASD cutoff on the AQ. Thus, none showed any sign, according to self-report, of manifesting significant ASD-like traits.

Second, a relative or long-standing friend of each participant completed a prepublication version of the Social Responsiveness Scale, Second Edition (SRS-2; Constantino & Gruber, 2012). Scores on this detailed questionnaire provide a valid and reliable indicator of participants’ social and communicative abilities. Only three participants with ASD missed the ASD cutoff on the SRS-2 (raw score ≥ 60). All comparison participants scored below the ASD cutoff on the SRS-2. Thus, none showed any sign, according to relatives/friends, of manifesting significant ASD-like traits.

In addition, 19 of 27 participants with ASD (the remaining 10 participants in the group were unwilling to take part in the assessment) also completed the Autism Diagnostic Observation Schedule–Generic (ADOS; Lord et al., 2000). The ADOS is a semistructured, standardized assessment of social interaction, communication, play, and imaginative use of materials, and is frequently used in the diagnostic assessment of ASD. ADOS assessments were administered by fully trained individuals who had achieved at least 80% reliability with the developers of the instrument. All participants with ASD who completed the ADOS (and all those who had missed the AQ and SRS-2 cutoffs) met the ASD cutoff (≥7 points).

Using the Wechsler Abbreviated Scale of Intelligence (WASI; Wechsler, 1999), the groups were matched closely for verbal and nonverbal ability. The groups were also matched closely for chronological age. Importantly, all effect sizes associated with group differences in baseline characteristics of age and IQ were negligible/small. Participant characteristics are presented in Table 1.[Table-anchor tbl1]

### Test and Procedures

#### Experimental (episodic memory, episodic future thinking, and scene construction) task

Each participant was tested individually in a quiet room, and sat opposite the experimenter. Participants were instructed that they would be asked to imagine or remember vivid scenes in their mind, based on a cue card that would set the scene. They would then have to describe this mental representation to the experimenter in as much detail as possible. Before commencing the task, an example cue card (“Imagine you’re sitting on a bench in a park. Describe the scene in as much detail as possible”) was given by the experimenter, who also provided a model answer. It was highlighted to participants that the description given by the experimenter was multimodal, containing not only visual details but also smells, sounds, and so forth. Each participant was asked to produce a description for 12 scenarios, split into three separate conditions: past events (last week, last birthday, last time they went shopping), future events (this weekend, next Christmas, next time they see a friend or relative), and fictitious scenes (beach, museum, pub, ship, market, forest). Trials were blocked by condition and, across participants, the three conditions were presented in counterbalanced order. For all descriptions in the future events and fictitious scenes conditions, participants were explicitly instructed not to recount an actual memory, or any part of one, but rather to generate a specific novel episode/scene in their mind. In contrast, for the past events condition, participants were told they must recall and describe a real personally experienced episode. Participants’ descriptions were audio recorded for later transcription and coding.

On each trial, a cue card was placed on the table in front of the participant, detailing a short description of the scenario to be described (e.g., “Standing by a small stream, somewhere deep in a forest”). This card remained on the table throughout each trial, to act as a cue to the participant and remind them of the scenario if necessary. The experimenter read aloud this scenario and asked the participant to produce a vivid multimodal description of the experience and surroundings, using all of their senses.

A probing protocol was followed (as outlined in Hassabis, Kumaran, & Maguire, 2007), such that general prompts were given if a description could not be provided or lacked detail (e.g., “Tell me more about the scene”). If a participant became fixated on one aspect of the scene, they were encouraged to move on, and if they provided poor detail, they were asked to elaborate further. Only such general prompts were given, and the experimenter was careful not to lead the participant or introduce any aspect or detail that had not been mentioned by the participant previously. Participants were encouraged to continue with their descriptions until the account came to a close, or until they were unable to elaborate any further.

Following each trial, participants completed a questionnaire rating their response to the cue on a series of elements including how salient the imagined/remembered scene/episode was and how much of a sense of presence they had when imagining/remembering the scene/episode. They were also presented with a series of 12 statements that were designed to gauge how integrated or fragmented their description was thought to be (e.g., “It was not so much a scene as a collection of images”; “I could see it as one whole scene in my mind’s eye”). Participants were asked to select those statements from the series that most applied to their imagined/remembered scene/episode.

#### Scoring

Each description was transcribed from audio recordings by an independent transcriber (who was blind to group status and to the aims of the study) and the transcription was subsequently coded according to the detailed guidelines provided by Hassabis, Kumaran, & Maguire (2007). This coding was carried out by an independent rater who was also blind to group status and to the aims of the study (she had access only to written transcripts—she was not involved in any testing and did not have access to any audio recordings). To assess the reliability of the judgments provided by the main rater, a second coder rated a randomly selected subset (*n* = 30; 54%) of the transcripts (see reliability values below in the “Description content” and “Independent quality ratings” subsections).

For each description, a composite “experiential index” score was calculated, ranging from 0 to 60. This provides the key overall measure of how rich and detailed each description was. The composite score was calculated by combining the following four subcomponent scores.

##### Description content

Within each description, statements were coded as belonging to one of four categories: spatial references, entity presence, sensory description, or thought/emotion/action. On the basis of pilot studies, Hassabis, Kumaran, & Maguire (2007) argued that the production of seven instances per category in each description should be considered a reflection of optimal performance. Thus, the total for each category was a maximum score of 7. This yielded a score out of 28 for each description, as an indication of content quality. Interrater reliability for description content across the four categories was high, Cronbach’s α = .97.

##### Participant questionnaire ratings

Ratings from two of the questions that participants completed in the postdescription questionnaires were included in the experiential index. First, their sense of presence in the description was rated on a scale of 1 to 5 (*did not feel like I was there at all* to *strongly felt like I was really there*). Second, the perceived salience of the imagined scene was also rated on a scale of 1 to 5 (*couldn’t really see anything* to *extremely salient*). Each of the scores was on a scale of 1 to 5, later rescaled to scores from 0 to 4 (following Hassabis, Kumaran, & Maguire, 2007).

##### Spatial coherence index

This score was calculated from the responses participants made to the 12 statements on the postdescription questionnaires. Participants were required to tick as many of the statements as they thought applied to the imagined/remembered scene/episode they had just generated. Eight of the statements were “integrated” and suggested that the description was a continuous whole (e.g., “I could see it as one whole scene in my mind’s eye”), and four indicated that the scene was more “fragmented” (e.g., “I could see individual details, but it didn’t all fit together as a whole scene”). For each integrated statement that was selected, one point was awarded. For each fragmented statement that was selected, one point was subtracted. When totaled, these scores ranged between −4 and +8. This score was then normalized, to give a spatial coherence index between −6 and +6, with the coherence of the description increasing as the score increased.

##### Independent quality ratings

Each description was rated out of 10 for its general quality, based on the extent to which it reflected a specific and detailed idea, and to which it reflected a vivid picture of the experience for the rater themselves. A score of 0 indicated that the description lacked any detail or vivid experience, and a score of 10 was assigned if the description was richly detailed and evoked a vivid sense of experiencing. These scores were then rescaled to a score of between 0 and 18, by multiplying by a factor of 1.8. Interrater reliability for the independent quality ratings was high, Cronbach’s α = .94.

The experiential index was calculated by adding up each of these subcomponent scores: description content (between 0 and 28) + sense of presence (between 0 and 4) + perceived salience (between 0 and 4) + spatial coherence index (between 0 and 6) + independent quality rating (between 0 and 18).

The final experiential index score thus ranged between 0 (representation lacked detail and vivid experiencing) and 60 (richly detailed and experienced).

In addition to coding the elements included in the Hassabis, Kumaran, & Maguire (2007) content score, we also recorded the number of temporal terms (e.g., yesterday, tomorrow, times of year) used by participants in their descriptions. This allowed us to assess the extent to which participants really were engaging in mental time travel during the past and future event conditions of the task. If participants were engaging in episodic memory to remember experiences *from the past* in the past event condition, and engaging in episodic future thinking to imagine events that may occur *in the future* in the future event condition, but imagining atemporal scenes in the fictitious scene condition, then significantly fewer temporal references should be made in the fictitious scene condition than in either of the other conditions.

### Narrative Control Task

The book *Frog, Where Are You?* (Mayer, 1969) was used to elicit narratives. This is a 24-page picture book that has been used in several studies of narrative ability among individuals with developmental disorders, including ASD (see Norbury & Bishop, 2003). The book is based around the adventures of a boy and his pet dog when they go looking for his pet frog after the frog escapes during the night.

The participant was shown the front cover of the book and asked to confirm that they had not encountered the book before. No participant was familiar with the book. They were told that it was a picture book and were instructed to look at the pictures and tell the story. Participants were informed that the experimenter had never seen the book before, and therefore they needed to be as clear as possible while telling the story. To eliminate memory demands, participants looked at each picture and turned the pages as they told the story aloud. The room set up was such that the experimenter could not see the pictures as the story was being told, to reinforce the experimenter’s lack of knowledge of the storyline. The experimenter did not interrupt the participant once they began their narrative and all narratives were audio recorded for later transcription and coding.

#### Scoring

Each narrative was transcribed by an independent transcriber. The narrative scripts were coded by one rater who was blind to participant diagnosis. A second rater then coded a randomly selected subset (*n* = 14; 25%) of the narrative scripts. The narratives were scored on three key dimensions: length of the narrative, global structure of the narrative, and number of relevant semantic details given.

##### Length (unbounded score)

The overall length of the narrative in words was calculated after the deletion from the transcript of repetitions and disfluencies, such as “ums” or “errs.”

##### Global structure (from 0 to 6)

The global structure measure was included as an index of the participant’s understanding of the causal structure of the story (following the procedure adopted by Reilly, Bates, & Marchman, 1998; see also Norbury & Bishop, 2003). Two points were given for the *initiating event* if the participant included in their narrative details of the frog escaping (1 point) and the boy looking for the frog in his room (1 point). Two points were given for mentioning two or more of the events that occur during the *search* for the frog (1 point for each episode, up to a total of 2 points). Two final points could be awarded if the *resolution* was narrated correctly; the boy eventually finds his frog (1 point) and takes the frog home with him (1 point). Interrater reliability for the global structure score was high, Cronbach’s α = .94.

##### Semantic score (from 0 to 102)

This score provides an indicator of the amount of relevant detail included in participants’ narratives. The scoring procedure developed by Norbury and Bishop (2003) was adopted, and participants were scored on how accurately and fully they included a list of 51 story elements in their narrative. Participants were given a score of 2 for every story element that they accurately included in their narrative, with a score of 1 given for an element they included inaccurately or only partially elaborated (for detailed guidance, and a list of the 51 story elements; see Norbury & Bishop, 2003). Interrater reliability for the semantic score was high, Cronbach’s α = .99.

### Theory of Mind Task

The animations task requires participants to describe interactions between a large red triangle and a small blue triangle, as portrayed in a series of silent video clips. Eight clips (taken directly from Abell et al., 2000) were employed. On the one hand, four of the clips were designed such that explanation of the triangles’ behavior required the attribution of epistemic mental states, such as belief, intention, and deception (“seducing,” “coaxing,” “surprising,” and “mocking” animations). On the other hand, four other clips were designed such that explanation of the triangles’ behavior required the attribution of physical agency, without reference to complex mental states (“fighting,” “following,” “chasing,” and “dancing” animations). Following Heider and Simmel (1944), Abell et al. referred to these clips as involving “goal-directed” action (e.g., copying, chasing, following). Although these are certainly goal states, and hence could be considered mental states (albeit nonepistemic mental states), the explanation of the triangles’ behavior in these clips requires only a focus on the actions displayed by the characters, rather than on the underlying mental states that cause the actions. For example, to describe two characters as “kissing” does not require any understanding of the mental states underlying that behavior, although it does require the perception of the characters as animate agents. It is for this reason that we refer to these clips as comprising a “physical” condition, which we contrast with the mentalizing condition described previously.

Each clip was presented to participants on a computer screen. To familiarize participants with the task, two practice animations were shown before the experimental stimuli (one physical and one mentalizing). Participants were asked to describe the behavior displayed in each of these clips, and experimenter feedback was given after each description. For the experimental animations, participants were asked to “watch the clip and give me a running commentary about how the triangles are interacting.” For the experimental trials, a digital audio recording of participants’ responses was made for later transcription. No feedback was given on the experimental trials. The order in which the experimental clips were presented was counterbalanced across participants.

#### Scoring

Each description was transcribed by an independent transcriber. Participants’ descriptions were scored on the basis of scoring criteria outlined in Abell et al. (2000; see their Appendix A for detailed scoring criteria). Participants’ descriptions of each animation were given a score of 0, 1, or 2 according to their level of accuracy. Accuracy was defined as the extent to which the participant’s description captured the intended meaning of the animation. Thus, the score achievable in each condition (mentalizing/physical) was between zero and eight. Each description was scored by an independent rater who was blind to group status. A second rater then coded each of the transcripts. Interrater reliability for scores across each of the eight animations was high, Cronbach’s α = .99.

### Statistical Analyses

Results were analyzed using the statistical software package SPSS (Version 19). A standard alpha level of .05 was used to determine statistical significance for all analyses. All reported significance values are for two-tailed tests.

In the first instance, group differences on the two background tasks—the theory of mind animations and narrative control tasks—were explored using univariate and multivariate ANOVAs, respectively. Next, the data from the main experimental (episodic memory, episodic future thinking, and scene construction) task were analyzed using a series of univariate ANOVAs and *t* tests. Where ANOVAs were used, we report the corresponding partial η^2^ values as a measure of effect size. Partial η^2^ values of ≥.01 indicate small effects, values ≥.06 indicate medium effects, and values ≥14 indicate large effects (Cohen, 1969). Where *t* tests were used, we report the corresponding Cohen’s *d* value as a measure of effect size. Cohen’s *d* values of ≥.0.20 indicate small effects, values of ≥0.50 indicate medium effects, and values of ≥0.80 indicate large effects (Cohen, 1969). Finally, in order to explore the relationship between specific aspects of performances on the main experimental task and performances on the theory of mind task, a series of Pearson’s correlation analyses were conducted.

## Results

### Background Task Performance

#### Animations task

In the mentalizing condition, participants with ASD scored a mean of 3.52 (*SD* = 1.87) and comparison participants scored a mean of 4.62 (*SD* = 1.63). In the physical condition, participants with ASD scored a mean of 5.76 (*SD* = 1.39) and comparison participants scored a mean of 6.90 (*SD* = 1.14). A 2 (group: ASD, comparison) × 2 (condition: mentalizing, physical) mixed design ANOVA revealed a significant main effect of condition, *F*(1, 52) = 84.57, *p* <.001, partial η^2^ = .62, reflecting superior performance in the physical condition than in the mentalizing condition. There was also a significant main effect of group, *F*(1, 52) = 11.05, *p* = .002, partial η^2^ = .18, reflecting poorer overall performance among participants with ASD than among comparison participants. However, the interaction between group and condition was not significant, *F*(1, 52) < 0.01, *p* = .94, partial η^2^ < .01. Thus, the performance of participants with ASD was as diminished in the physical condition of the task as it was in the mentalizing condition of the task.

#### Narrative control task

Table 2 shows the average score on each measure of narrative ability among ASD and comparison participants.[Table-anchor tbl2]

An overall multivariate analysis of these three measures, with group (ASD, comparison) as the between-participants variable, revealed a nonsignificant main effect of group, *F*(3, 52) = 1.58, *p* = .21, partial η^2^ = .08. Table 2 highlights that group differences on each of the measures individually (i.e., global structure, semantic structure, narrative length) were associated with small effect sizes in each case. Thus, there was no evidence that participants with ASD were markedly less able than comparison participants to provide a narrative of stimuli they experienced in the external environment. As such, any group differences in the ability to provide descriptions of memories, imagined future events, or imagined fictitious scenes are unlikely to be the consequence of basic difficulties with narration among participants with ASD.

### Experimental Task Performance

#### Composite experiential index score

**Figure 2 fig2:**
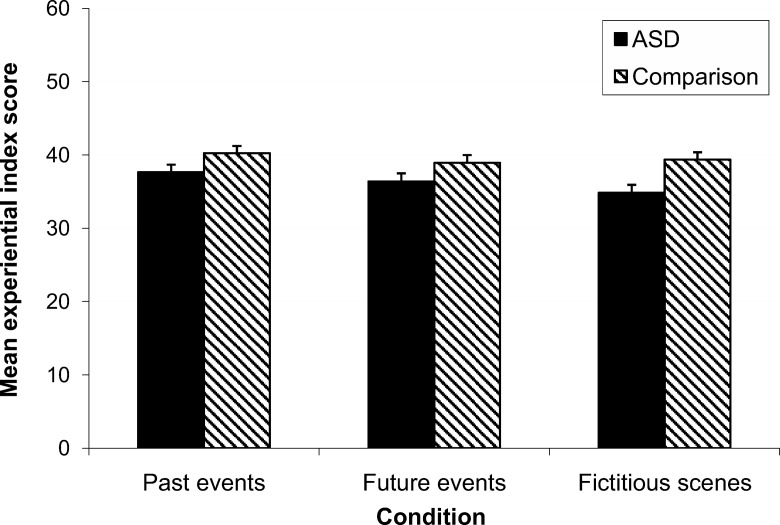
Mean experiential index score achieved by ASD and comparison participants in each condition of the experimental task (error bars represent 1 *SEM*).

Figure 2 shows the mean experiential index scores achieved by ASD and comparison participants in each condition of the experimental task.[Fig-anchor fig2]

A 3 (condition: past events, future events, fictitious scenes) × 2 (group: ASD, comparison) mixed design ANOVA was conducted, with experiential index score as the dependent variable. This revealed a significant main effect of condition, *F*(2, 108) = 4.32, *p* = .02, partial η^2^ = .07. Paired-samples *t* tests showed that, within the combined participant groups, descriptions of past events (*M* = 39.00, *SD* = 5.38) were associated with a higher experiential index score than descriptions of either imagined future events (*M* = 37.70, *SD* = 5.83), *t*(55) = 2.27, *p* = .03, *d* = 0.30, or fictitious scenes (*M* = 37.19, *SD* = 5.89), *t*(55) = 2.56, *p* = .01, *d* = 0.35. The experiential index score for descriptions of imagined future events did not differ significantly from that for descriptions of fictitious scenes, *t*(55) = 0.79, *p* = .43, *d* = 0.11.

Crucially, there was also a significant main effect of group, *F*(1, 54) = 6.37, *p* = .02, partial η^2^ = .11. Across all conditions, the experiential index score was significantly lower among participants with ASD than among comparison participants, *t*(54) = 2.52, *p* = .02, *d* = 0.68. Finally, there was no significant interaction between group and condition, *F*(2, 108) = 1.50, *p* = .23, partial η^2^ = .03. Thus, the experiential index score was higher among comparison participants than among participants with ASD to the same extent within each condition.

#### Experiential index subcomponents

Table 3 shows the mean experiential index scores, plus the mean scores for each of its components collapsed across condition (past events, future events, and fictitious scenes), among ASD and comparison participants.[Table-anchor tbl3]

To investigate group differences in performance on each subcomponent of the experiential index, we conducted a series of further 3 (condition: past events, future events, fictitious scenes) × 2 (group: ASD, comparison) mixed design ANOVAs that included, respectively, spatial coherence score and overall quality rating, as well as each of the participants ratings (sense of presence; perceived salience) and each content score (spatial references; entities present; sensory details; thoughts/emotions/actions). In all but one of these analyses, there was no hint of any significant interaction between group and condition (all *p*s > .18; all partial η^2^s ≤ .03). In the analysis concerning the entities present subcomponent of the content score, the interaction between group and condition approached but did not reach statistical significance (*p* = .06, partial η^2^ = .06). Given the lack of any significant Group × Condition interaction effects, for the sake of brevity, we follow Hassabis, Kumaran, & Maguire (2007) and Maguire, Vargha-Khadem, and Hassabis (2010) in reporting only the main effect of group in each analysis (i.e., collapsed across past events, future events, and fictitious scenes conditions). The *F*, *p,* and Cohen’s *d* values for each main effect of group are reported in Table 3.

In summary, with respect to participant ratings, individuals with ASD manifested a significantly reduced sense of presence in their imagined/remembered events/scenes. Participants with ASD also reported that the imagined/remembered events/scenes were significantly less salient than did the comparison participants. Spatial coherence scores were also significantly lower among ASD than comparison participants, indicating that individuals with ASD experienced their mental representations as more fragmented and less coherent than did comparison participants.

In terms of the objective, independently rated content scores, there were no significant differences between the groups in terms of number of spatial references, number of entities present, or the number of sensory details in the descriptions. The difference between the groups in the number of thoughts/emotions/actions present in descriptions was close to statistical significance (*p* = .06, partial η^2^ = .06). Finally, the other objectively rated score—for the overall quality of descriptions provided—was significantly lower among participants with ASD than among comparison participants. Thus, participants with ASD produced descriptions that were significantly less vivid and provided less evidence of event-specificity (i.e., that the episode/scene described was specific and unique) than did comparison participants.

Here, it is important to highlight, again, that the descriptions provided by participants were blind-coded by a rater who provided highly reliable judgments. Thus, it is unlikely that the reduced quality of descriptions provided by participants with ASD was merely a consequence of rater bias. Moreover, the ability to narrate external stimuli was unimpaired among participants with ASD, suggesting that the reduced quality of descriptions provided by participants with ASD genuinely reflected impoverished ability to imagine/remember events/scenes *in mind*. However, to be absolutely stringent, we re-ran the analysis concerning overall quality of descriptions, but this time included narrative ability as a covariate. For the purpose of this ANCOVA, we calculated standardized (*Z*) scores for each of the three indices of narrative ability (length, global structure, and semantic score). We then took an average of these three scores and used this as the covariate. The data met the assumptions for ANCOVA. The effect of group remained significant in the ANCOVA, *F*(2, 53) = 4.27, *p* = .04, partial η^2^ = .07,^3^ thus confirming the earlier finding that group differences in the overall quality of descriptions were independent of general narrative ability.

#### Temporal references

**Figure 3 fig3:**
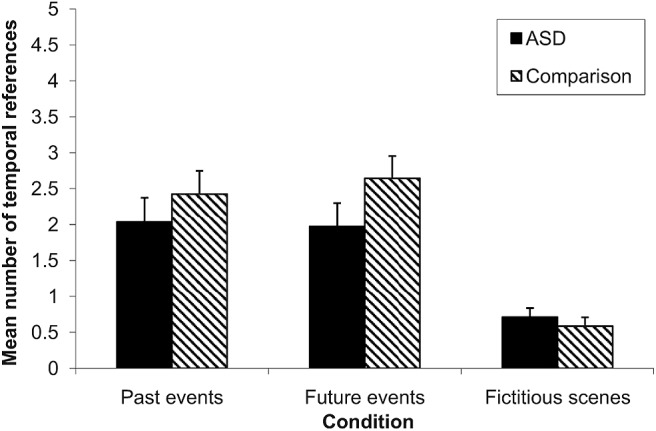
Mean number of temporal references made by ASD and comparison participants in each condition of the experimental task (error bars represent 1 *SEM*).

Figure 3 shows the mean number of temporal references made by ASD and comparison participants in each condition of the experimental task.[Fig-anchor fig3]

A 3 (condition: past events, future events, fictitious scenes) × 2 (group: ASD, comparison) ANOVA was conducted with mean number of temporal references in participant descriptions as the dependent variable. This revealed a significant main effect of condition, *F*(2, 108) = 38.68, *p* <.001, partial η^2^ = .42. Within-participants *t* tests showed that descriptions of fictitious scenes contained significantly fewer temporal references than descriptions of either actual past events, *t*(55) = 7.70, *p* <.001, *d* = 1.29, or imagined future events, *t*(55) = 7.92, *p* <.001, *d* = 1.26. The number of temporal references contained in descriptions of past events did not differ from the average number contained in descriptions of imagined future events, *t*(55) = 0.37, *p* = .71, *d* = 0.05. The main effect of group was nonsignificant, *F*(1, 54) = 1.08, *p* = .30, partial η^2^ = .02. The interaction between group and condition was also nonsignificant, *F*(1, 54) = 1.78, *p* = .17, partial η^2^ = .03. Thus, there were no differences between the groups in terms of either the overall frequency of temporal references made, or in the distribution of temporal references across conditions.

### Correlation Analysis

We conducted a series of partial correlation analyses to explore the association between theory of mind task performance and performance on the experimental task. With regard to variables from the experimental task, we employed only the experiential index score in the correlation analysis. It was an *a priori* decision to focus only on this key composite score, given the sheer number of correlations that would need to be computed if we included scores on each subcomponent of the task in addition. For the purpose of the correlation analyses, we collapsed participants’ scores across the past events and future events conditions to produce an average “mental time travel” score. As discussed earlier, our rationale was that performance in these two conditions required self-projection and self-related processing skills over and above basic scene construction ability. Basic scene construction ability was, of course, measured by performance in the fictitious scenes condition of the experimental task.

#### Episodic memory/future thinking and theory of mind

In the first correlation analysis, we explored the association between the mental time travel score from the experimental task and the mentalizing score from the animations task, controlling for performance in the fictitious scenes condition of the experimental task and performance in the physical condition of the animations task. We argue that this provides a pure test of the relation between (temporal) self-projection ability and theory of mind, independent of basic scene construction ability *and* independent of general (non-mentalizing) demands inherent in the animations task. Among the whole sample of participants, the association was positive and significant, *r* = .29, *p* < .05 (and among each group independently, the strength of the association was almost identical: among ASD participants, *r* = .29; among comparison participants, *r* = .33).^4^

#### Scene construction and theory of mind

In the second analysis, we explored the association between performance in the fictitious scenes condition of the experimental task and the mentalizing score from the animations task, controlling for performance in the physical condition of the animations task. As previously, we wanted to gain a relatively pure measure of the relation between scene construction ability and theory of mind ability independent of general (nonmentalizing) demands inherent in the animations task. Note that in this analysis, which is a reverse of the first analysis, the mental time travel score of the experimental task should not be controlled for in the manner that we controlled for fictitious scenes score in the previous analysis. This is because the mental time travel score is accounted for by *both* scene construction and (temporal) self-projection abilities. Thus, controlling for the mental time travel score would partial out some variance in theory of mind ability that is explained by scene construction, which would defeat the purpose of investigating the extent to which scene construction and theory of mind are associated. Among the whole sample of participants, the association was positive but nonsignificant, *r* = .14, *p* = .33 (and among each group independently, the strength of the association was almost identical: among ASD participants, *r* = .10; among comparison participants, *r* = .09).

A Fisher’s *Z* test of the difference in the size of the coefficients produced in the first and second correlation analyses was nonsignificant, *Z* = 0.81, *p* = .42. Thus, although the association between self-projection ability and theory of mind was statistically significant, whereas the association between scene construction ability and theory of mind was not significant, the correlations themselves were not significantly different in size.

## Discussion

It is well established that individuals with ASD have a selective diminution of episodic memory (e.g., Bowler et al., 2000). Given that the ability to remember previously experienced episodes is thought to depend on the same underlying cognitive and neurobiological mechanisms/processes as the ability to imagine (or mentally “pre-experience”) self-relevant future events, a diminution of episodic future thinking should also be evident among people with ASD. However, previous studies of episodic future thinking among individuals with ASD (as well as among individuals with developmental amnesia) have produced mixed results, providing a significant challenge to theories that posit an inherent link between episodic memory and episodic future thinking. However, the current results arguably provide the clearest evidence to date, regarding episodic future thinking abilities among people with ASD and they suggest that this ability is, indeed, diminished to the same extent as episodic memory in this population.^5^

In line with the findings of Lind and Bowler (2010), participants with high-functioning ASD in the current study produced descriptions of imagined future events (and of personally experienced past events) that were of significantly lower quality (i.e., less specific, less episodic) than did comparison participants. However, the current study has two advantages over that conducted by Lind and Bowler. First, the current study involved twice as many participants (*n* ≥ 27 per group, which is large compared with most studies of ASD) as Lind and Bowler’s study, which serves to increase confidence in the reliability of the findings. Second, the current study included a narrative control task, which allowed us to confirm that diminished experimental task performance was not merely the result of reduced general narrative ability among participants with ASD. Yet despite these improvements on Lind and Bowler’s study, the experimental results of each study were notably similar (with the effect sizes for group differences being medium-to-large in each study), suggesting that Lind and Bowler’s results were, in fact, representative, and that episodic future thinking is reliably impaired among people with ASD, independent of general narrative ability.

Arguably the most important finding in the current study, however, was that in addition to manifesting a diminution of the abilities to mentally re-experience personal episodes from the past (episodic memory) and the ability to mentally pre-experience self-relevant personal episodes that one might plausibly experience in the future (i.e., episodic future thinking), individuals with ASD also showed a diminished ability to imagine atemporal, *non-self-relevant* fictitious scenes—that is, scene construction was impaired. This is the first study that we know of to have explored scene construction ability among individuals with ASD, and this supports the idea that a diminution of the ability to bind together elements of a scene in mind may be a major underlying cause of episodic memory and episodic future thinking impairments in ASD.

Importantly, there was evidence in the current study to support the theory put forward by Hassabis, Kumaran, & Maguire (2007) that the past and future events conditions of the experimental task involve self-projection in time/self-related processing in addition to basic scene construction ability. First, significantly more spontaneous temporal references were made in the past and future events conditions than in the fictitious scenes condition. This implies that individuals from both groups were spontaneously engaging in some form of “mental time travel” when imagining possible future episodes and recalling previously experienced episodes, but not when they were imagining scenes in the fictitious scenes condition. Second, performance in the past and future events conditions was associated significantly with theory of mind ability, whereas performance in the fictitious scenes condition was not. Specifically, performance in the past and future events conditions was associated significantly with theory of mind ability *independent* of scene construction ability. This suggests that it was the self-projection/self-related processing component of the past and future events conditions specifically that drove the association with theory of mind (cf. Buckner & Carroll, 2007). This is consistent with findings from neuroimaging studies that regions of the brain traditionally implicated in theory of mind are significantly more active during episodic future thinking than during non-self-related fictitious scene construction (see Hassabis, Kumaran, & Maguire, 2007).

The fact that the performance of participants with ASD in this study was equally diminished in all conditions (including the fictitious scenes condition) suggests that an underlying difficulty with basic scene construction may be necessary and sufficient to cause episodic memory and episodic future thinking deficits in ASD. Certainly, any difficulties with self-projection/self-related processing did not impair the episodic future thinking and episodic memory performance of participants with ASD over and above impairments caused by difficulties with basic scene construction. Thus, the current results support the notion that hippocampal dysfunction might contribute to episodic memory and episodic future thinking impairments in ASD (e.g., Bowler et al., 2011).

Of course, our study did not incorporate a neuroimaging element and, as such, we cannot draw firm *conclusions* about the brain basis/neuroanatomical locus of scene construction deficits in ASD. However, given the consistent finding from the imaging literature that scene construction depends upon the functioning of the hippocampus, coupled with the finding here that scene construction is diminished in ASD, we believe it is possible to *infer* that our findings add weight to suggestions that abnormalities of the hippocampus may impair the binding process necessary for encoding, storage, and/or retrieval of episodic information among people with ASD. Although neuroimaging studies of ASD produce inconsistent and unreliable results, several studies have demonstrated abnormal hippocampal volume in ASD (see Stanfield et al., 2008). In terms of functional neuroimaging, only one relevant study of ASD has been conducted, to our knowledge. Gaigg, Bowler, Ecker, Calvo-Merino, and Murphy (2010) found atypical hippocampal activation during an episodic binding task. The findings from the current cognitive-experimental study are in keeping with these findings.

The observation of scene construction deficits in ASD is also consistent with the notion that perceptual processing among people with this disorder tends to be characterized by weak central coherence. In the current study, the spatial coherence score on the experimental task was significantly lower among ASD than comparison participants, indicating greater fragmentation and less coherence of mental representations. That people with ASD have difficulty binding together elements of a scene *in their mind* might well be related to a corresponding difficulty with binding together elements of a scene *in the environment*. Therefore, future studies of ASD might usefully explore any potential relation between scene construction ability and perceptual processing style.

In summary, this study confirms earlier findings of diminished episodic future thinking (and episodic memory) among people with ASD (cf. Lind & Bowler, 2009). More importantly, it extends previous studies of this ability not only by including a substantially larger sample but also by controlling for the effects of general narrative ability on experimental task performance. Given that previous studies of episodic future thinking in ASD relied on participants providing verbal descriptions of complex mental representations, any apparent deficit in episodic future thinking may have been due merely to difficulties with narration, rather than due to limitations in the component processes that underpin episodic future thinking. The current study strongly suggests that this is not the case and that diminished scene construction ability is a primary determinant of episodic memory/episodic future thinking impairment among people with ASD. Indeed, the current study is the first to explore basic scene construction ability in ASD and it is significant that impairments were observed in this population.

Given that the current data were obtained from an adult sample, we can only speculate regarding the developmental origins of the observed deficit in scene construction. However, it seems reasonable to suppose that it is present from early in development, given that impairments in episodic memory, which are thought to depend on scene construction ability, have previously been demonstrated among samples of children with autism (e.g., Bruck, London, Landa, & Goodman, 2007; Lind & Bowler, 2009). The impairments observed in this study are likely to have significant clinical implications (and particularly so, if they are long-standing). Episodic future thinking is thought to be essential for flexibility of thought and action because it enables one to simulate and predict future scenarios, thereby allowing one to plan and select the optimal course of action (see Suddendorf & Corballis, 2007). It follows that difficulty in acting with the future in mind may result in overdependence on routinized, inflexible patterns of behavior. Thus, impairments in prospection may potentially help to explain why individuals with ASD characteristically exhibit restricted, repetitive, and stereotyped patterns of behavior. Hence, it may be profitable for future research to explore whether training scene construction ability could serve to remediate limitations in episodic future thinking.

## Figures and Tables

**Table 1 tbl1:** Participant Characteristics (Means and Standard Deviations)

	ASD (*n* = 27)	Comparison (*n* = 29)	*t*	*p*	Cohen’s *d*
Age (years)	35.46 (13.23)	33.25 (16.15)	0.56	.58	0.15
VIQ	111.59 (15.08)	112.97 (12.06)	0.38	.71	0.10
PIQ	109.96 (16.21)	113.24 (12.33)	0.86	.40	0.23
FSIQ	112.37 (16.36)	114.07 (11.01)	0.45	.65	0.12
AQ	34.44 (8.78)	12.52 (5.41)	11.16	<.001	3.01
SRS-2^a^	94.68 (30.81)	18.41 (19.07)	10.63	<.001	2.98
ADOS-G^b^	11.05 (2.88)	—	—	—	—
*Note.* ASD = autism spectrum disorder; VIQ = verbal IQ; PIQ = performance IQ; FSIQ = full-scale IQ; AQ = Autism-spectrum Quotient; SRS-2 = Social Responsiveness Scale, Second Edition; ADOS-G = Autism Diagnostic Observation Schedule–Generic.					
^a^ Based on 25/27 participants with ASD and 27/29 comparison participants. ^b^ Based on 19/27 participants with ASD.					

**Table 2 tbl2:** Mean (SD) Scores on Each Measure of Performance on the Narrative Control Task

	ASD (*n* = 27)	Comparison (*n* = 29)	Cohen’s *d*
Narrative length (number of words)	751.44 (374.90)	729.55 (330.15)	0.09
Global structure (0–6)	5.52 (0.64)	5.76 (0.52)	0.42
Semantic score (0–102)	68.78 (14.66)	74.57 (13.36)	0.42
*Note.* ASD = autism spectrum disorder.			

**Table 3 tbl3:** Mean (SD) Experiential Index Score, and Scores on Each of Its Subcomponents Collapsed Across Conditions of the Experimental Task

	ASD (*n* = 27)	Comparison (*n* = 29)	*F*^a^	*p*^a^	Cohen’s *d*^a^
Experiential index score (0–60)	36.30 (5.38)	39.51 (4.09)	6.37	.02	0.68
Subcomponents					
Content					
Spatial references (0–7)	2.06 (1.63)	1.65 (1.23)	1.15	.29	0.29
Entities present (0–7)	6.96 (0.11)	6.98 (0.47)	1.42	.24	0.06
Sensory descriptions (0–7)	6.39 (0.73)	6.66 (0.45)	2.71	.11	0.46
Thoughts/emotions/actions (0–7)	5.75 (0.90)	6.19 (0.83)	3.61	.06	0.52
Participant ratings					
Sense of presence (0–4)	2.17 (0.74)	2.64 (0.53)	7.48	.01	0.75
Perceived salience (0–4)	2.32 (0.68)	2.70 (0.47)	5.98	.02	0.67
Spatial coherence index (0–6)	1.37 (2.07)	2.45 (1.49)	5.12	.03	0.60
Overall rating of description quality (0–18)	8.49 (2.32)	9.88 (1.98)	5.87	.02	0.65
*Note.* ASD = autism spectrum disorder.					
^a^ Associated with the between-group difference in scores (i.e., the main effect of group in Group × Condition ANOVAs).					

## Footnotes

1 An additional pilot study of future thinking in ASD was published by Jackson and Atance (2008). However, participants in the study were not matched for age or (as a result) IQ, making the reported between-groups differences in future thinking very difficult to interpret.

2 It should be noted that there is some ambiguity in the position that Hassabis and colleagues take with respect to the necessity of scene construction for theory of mind. Of the diverse cognitive functions underpinned by self-projection according to Buckner and Carroll’s (2007) account, theory of mind is possibly the least discussed by Hassabis and colleagues. Hassabis and Maguire (2007, p. 301) suggest that scene construction is necessary for episodic memory, episodic future thinking, navigation, daydreaming, imagination, “default mode” thinking, viewer replay, and vivid dreaming. The only cognitive function (of those they consider) that they argue does *not* require scene construction necessarily is theory of mind. Rather, with respect to theory of mind, they argue that any possible role for scene construction “depends on the precise nature of the task and the content operated on.” From this, one might infer that they believe that some specific theory of mind tasks require scene construction by virtue of extraneous (non-theory of mind) task factors. However, it appears from this paper and others that Hassabis and colleagues do not believe that theory of mind processing itself requires scene construction necessarily. On the basis of evidence from neuroimaging studies, Hassabis, Kumaran, and Maguire (2007, p. 14372) argue indirectly that theory of mind is supported specifically by self-projection rather than scene construction: “Activation in amPFC and PCC, however, was only observed during episodic memory recall, suggesting that these regions support functions that are specific to episodic memory over and above scene construction (Tulving, 2001). In fact the pattern of activation revealed by this contrast bears a striking resemblance to networks found to support … theory of mind (Kumaran & Maguire, 2005).”

3 As in the original ANOVAs, ANCOVAs using each of the *content scores* (spatial references, number of entities present in the descriptions, number of sensory details in the descriptions, and number of thoughts/emotions/actions present in descriptions) as dependent variables and average standardized narrative score as the covariate did not reveal any significant effect of group (all *p*s >.07).

4 Additionally, we explored the association between the separate past events and future events scores, respectively, and the mentalizing score from the animations task, in each case, controlling for performance in the fictitious scenes condition of the experimental task and performance in the physical condition of the animations task. The correlations for past events scores, *r* = .23, *p* = .10, and future events scores, *r* = .24, *p* = .08, were almost identical, and the same patterns held within the ASD group (past: *r* = .23, *p* = .30; future: *r* = .27, *p* = .21) and comparison group (past: *r* = .29, *p* = .14; future: *r* = .27, *p* = .17) when analyzed separately.

5 Of course, because our sample consisted of exclusively intellectually high-functioning adults with ASD, we cannot be certain whether the same deficits would be observed among intellectually low-functioning adults with ASD, or among children with ASD (regardless of level of intellectual functioning). However, in our view, if a study observes impairment among intellectually high-functioning adults with ASD, it is highly unlikely that a deficit would not be observed among younger and/or intellectually less able individuals with ASD (among whom capacity to develop compensatory strategies is significantly limited).
